# Ask autistic adolescents too: informant discrepancies and test-retest reliability in social behavior

**DOI:** 10.3389/fpsyt.2026.1819507

**Published:** 2026-09-21

**Authors:** Hunter Mattern, Glenn I. Roisman, K. Suzanne Scherf

**Affiliations:** 1 Department of Psychology, Penn State University, University Park, PA, United States; 2 Institute of Child Development, University of Minnesota, Minneapolis, MN, United States; 3 Department of Neuroscience, Penn State University, University Park, PA, United States; 4 Social Science Research Institute, Penn State University, University Park, PA, United States

**Keywords:** adolescence, autism, inter-rater reliability, parent-report, self-report, social functioning, social skills improvement system

## Abstract

Assumptions about autistic youths’ inability to accurately self-report their own behavior have limited what is known about adolescent-parent informant discrepancies. Across two randomized controlled trials, autistic adolescents (10–18 years) and their parents independently reported on adolescents’ social skills and problem behaviors approximately 2–3 months apart. Test-retest reliability was evaluated among control-condition dyads (N = 40). Adolescent reports demonstrated moderate test-retest reliability across both domains. There were no group differences in reporting of social skills. Regarding problem behavior, adolescents reporting lower scores were more likely to report an increase over time, and those with higher scores a decrease over time. Parent reports were moderately reliable. They reported a systematic decrease in problem behavior over time; however, parents reporting higher problem behavior overall were more likely to report an increase in these behaviors over time. Using baseline data from all dyads (N = 81), we observed poor association and agreement between adolescent and parent reports in both domains. Adolescents reported more social skills and fewer problem behaviors than did their parents; the social skills discrepancy widened as scores increased. These findings challenge the assumption that autistic adolescents are unreliable reporters of their own behavior and suggest that including their self-reports alongside parent reports may provide unique, clinically meaningful information about social functioning during adolescence.

## 1 Introduction

During adolescence, youth increasingly gain autonomy from parents and invest in friendships and romantic relationships with peers (1), leaving parents with fewer opportunities to observe their behavior across contexts and less access to their internal experiences. As a result, parents may become less reliable reporters, in part because adolescent behavior becomes more variable across contexts (e.g., home versus school), a pattern known as *situational specificity (*2). One solution is to invite adolescents to report on their own behavior as well. Interestingly, adolescent and parent reports are often discrepant (3). For example, in non-autistic samples, parent and child reports of behavioral problems and mental health increasingly diverge in adolescence (2, 3). Associations are stronger for externalizing behaviors than for internalizing symptoms (2, 4). These discrepancies are not fully explained by unreliable adolescent reporting (5) or invalid measurements (4). Rather, scholars have interpreted them to reflect meaningful information about adolescent development and mental health (2–4) using methodologies that disentangle measurement error and rater biases from unique rater knowledge (6).

In contrast, research on informant discrepancies in *autistic adolescents* is primarily focused on parent–teacher discrepancies (7), as autistic adolescents are far less frequently asked to provide self-reports. Longstanding assumptions that autistic youth lack the capacity for accurate self-reflection (8, 9) led some to discount adolescent reports as unreliable, random, and inaccurate (10, 11). This legacy constrains what the field has learned about the lived experiences and motivations of autistic adolescents, which may be critical for understanding their social behavior (12) and how their experiences differ from what parents observe and perceive in their child’s behavior. By excluding autistic adolescents as meaningful informants of their own behavior, the field has likely missed critical information that could enhance the validity of assessments about the very behaviors that are central to the condition of autism (13). Here, we center autistic adolescents’ self-reports of their own social skills and problem behaviors to evaluate whether and how they contribute to informant discrepancies with parent reports.

### 1.1 Autistic adolescent-parent informant discrepancies

In a meta-analysis of informant discrepancies in autistic youth and youth with intellectual disabilities, only 26% of effect sizes assessed parent-child informant discrepancies (7). Across studies, correlations between parent and child reports were in the poor to moderate range (*r* = .23 -.52). As in the literature with non-autistic youth, inter-rater discrepancies on the child’s social skills increase when reporting on adolescents (*r* = .21) compared to school-aged children (*r* = .40) (7).

Multiple factors likely contribute to these discrepancies, including the actual characteristic being measured, the context in which the characteristic is being measured, the biases of the informants, and error of the measures (13). For example, autistic adolescents report greater use of social skills than their parents attribute to them (14–20). This difference could reflect the actual use of social skills, range of contexts in which adolescents and their parents observe adolescent behavior, different biases toward rating adolescent behavior, and/or measurement error. A recent study challenged the explanation that limitations in theory of mind in autistic adolescents account for these discrepancies (20). Additional work determined that autistic adolescents’ self-reports are not random or uniformly inflated; instead, they are comparable to or lower than, non-autistic peers’ self-reports, and they predict performance on independent measures of social functioning (19–21). However, there is little work evaluating the relative contributions of measurement error and informant biases in understanding autistic adolescent-parent informant discrepancies. Furthermore, almost nothing is known about individual differences in the magnitude and direction of discrepancies across adolescent-parent dyads. For example, it is not clear whether in addition to social skills, autistic adolescents also perceive themselves to exhibit more problem behavior than do their parents or whether the direction of a discrepancy between adolescent and parent raters is contingent on factors like the strength of behavior/skills in the adolescent.

### 1.2 The current study

Here, we begin to address these gaps. Parents and autistic adolescents completed the Social Skills Improvement System (22) (SSIS), a widely used measure in non-autistic research that is also recommended for use in autistic samples (23). Participants were tested in two randomized controlled trials (RCT) at pre- and post-intervention assessments approximately 2–3 months apart. We assessed the stability of reporting in both autistic adolescents and their parents over time via test–retest reliability analyses among participants assigned to control conditions (to avoid potential intervention-related effects). Differences in test-retest reliability between adolescent and parent reports could provide important information about discordance in true score variance and measurement error (24). Next, we assessed adolescent–parent report discrepancies from data acquired during the pre-intervention session prior to randomization, which allowed us to combine the intervention and control groups and double our power to estimate inter-rater agreement. For both analyses, we assessed association (i.e., Pearson’s *r*), agreement (intraclass correlation), and consistent [i.e., systematic (25)] rater biases (t-tests). We also visualized agreement discrepancies in Bland Altman plots (25, 26), which helped to interpret individual differences in both the direction and magnitude of informant discrepancies across the full range of scores. Finally, we used linear regression to assess whether rater differences vary as a function of score range [i.e., proportional rater bias (25)].

## 2 Methods and materials

### 2.1 Participants

This study was approved by the Institutional Review Board at The Pennsylvania State University. Written informed consent from parents and written assent from adolescents was obtained prior to participation.

Participants included 81 autistic adolescents (10–18 years old) and their parent who participated in one of two RCTs designed to improve sensitivity to eye gaze cues as signals of non-verbal communication. One SAGA2 dyad was excluded because the adolescent did not complete a full domain of responses. No dyads participated in both RCTs. Full protocols are available at clinicaltrials.gov (SAGA1: NCT02968225, SAGA2: NCT03690661). Both RCTs included intervention and control groups. Participant demographics of the individuals contributing to each analysis are presented in **Table 1**. Participant demographics for each study are presented in **Supplementary Materials**.

**Table 1:** Demographic and clinical characteristics of participants contributing to each analysis.

Characteristic	Test-retest analyses (control participants)	Inter-rater analyses (all participants at T1)
*N*	40	80
Age in months at T1	163.9 (28.2)	167.0 (29.7)
Sex - N (%) male	36 (90%)	67 (84%)
IQ		
Full Scale IQ	99.7 (14.8)	100.8 (15.6)
Verbal IQ	97.6 (18.6)	97.9 (18.2)
Non-Verbal	101.2 (13.7)	103.0 (14.4)
Clinical scores		
ADOS	13.80 (4.2) (N = 20)	13.5 (4.6) (N = 39)
SCQ	20.6 (7.8) (N = 20)	20.3 (6.3) (N = 41)

Age, IQ, and clinical scores are presented as mean (standard deviation; SD). Sex is presented as the total number (percentage) of male participants; remaining participants identified as female except for one SAGA2 intervention participant who identified as non-binary. Clinical diagnostic characteristics differed across studies due to the COVID-19 pandemic; SAGA1 utilized in-person eligibility assessments with the ADOS-2, while SAGA2 eligibility assessments were conducted remotely using the Social Communication Questionnaire (SCQ) and clinician interview. SAGA1 and SAGA2 participants were independent samples with no overlap across studies. Only participants in the control conditions were included in the test-retest analyses. All participant-parent dyads who participated in the pre-intervention testing session (i.e., T1) for both studies were included in the inter-rater analyses.

Adolescents had a full-scale IQ (27) between 70-130, minimum second grade reading level (28), and a confirmed autism diagnosis. Diagnosis confirmation was established by a licensed clinician using the Autism Diagnostic Observation Schedule (2^nd^ ed.) (ADOS-2) (29) in SAGA1, or the Social Communication Questionnaire (SCQ) (30), a clinical interview, and the Autism Diagnostic Interview-Revised (ADI-R) (31) in SAGA2. Detailed inclusion and exclusion criteria can be found in the RCT protocols.

### 2.2 Measures

#### 2.2.1 Social skills improvement system

The SSIS is one of six measures that are recommended for measuring changes in social communication in autism, given its clinical relevance, psychometric properties, sensitivity to change, and reasonable burden (22, 23). It assesses two domains: social skills and problem behavior. The SSIS Student Report (ages 8-12) was administered to adolescents; it includes 46 social skills and 29 problem behavior items. Adolescents rate *how true each statement is for them (*22) on a 4-point Likert scale. Although participants ranged from 10–18 years, we used this version for all adolescent participants to measure social skills of our youngest participants and because the reading level of this version is compatible with the inclusion criteria (i.e., minimum second grade reading level). The SSIS Parent Report was administered to parents; it includes 46 social skills and 33 problem behavior items. Parents rated *how frequently each behavior occurs (*22) on a 4-point Likert scale. Higher scores indicate more behavior in each domain being assessed.

### 2.3 Procedure

#### 2.3.1 Study context

Social Games for Autistic Adolescents (SAGA1, SAGA2) were experimental, two-phase RCTs evaluating serious game-based interventions designed to improve autistic adolescents’ sensitivity to and understanding of eye gaze cues during social interactions. In both studies, adolescents were randomized to the intervention or control condition using a 1:1 ratio (stratified by gender and full-scale IQ). In SAGA1 (32), participants assigned to the intervention condition played a serious game incorporating social eye gaze training, while participants assigned to the control condition continued with treatment as usual. In SAGA2, participants were assigned to either an updated version of the intervention game or an active control game which replaced the social eye gaze training with visuospatial object-processing tasks.

#### 2.3.2 Survey administration

Adolescents and parents independently completed the SSIS at the pre- and post-intervention sessions that were separated by 2 (SAGA1) or 3 months (SAGA2). The SSIS was administered via paper-and-pencil in SAGA1 and electronically via Qualtrics on an iPad in SAGA2. Parents completed the SSIS while adolescents underwent eye-tracking and behavioral testing. Adolescents completed the SSIS following testing in a separate room from parents. Research assistants read aloud items and answers, selected designated responses, or let participants read and respond themselves, depending on the level of support requested.

### 2.4 Analytic plan

All analyses were conducted using IBM SPSS Statistic version 31.0 (33). SSIS Social Skills (SS) and Problem Behaviors (PB) subscales were calculated separately for each informant (adolescent, parent), timepoint (pre-intervention, post-intervention), and study (SAGA1, SAGA2). Raw scores were converted to standardized scores (*M* = 100, *SD* = 15) prior to analyses. Participants who failed to provide complete responses on at least one domain within a time point were excluded from the analyses (N = 1 in SAGA 2). Missing data from adolescents or parents within a single domain (per session) were imputed using linear interpolation (N = 3 SAGA1 parents). Distributions of scores were evaluated for normality prior to analyses. To maximize statistical power, we collapsed participants across studies; additional study specific analyses are reported in **Supplementary Materials**.

#### 2.4.1 Test-retest reliability

Test-retest reliability was evaluated *using only the control condition* dyads. We computed Pearson’s correlation coefficients (*r*) to assess *linear associations* between pre- and post-intervention scores for adolescents and parents separately in each domain. We computed intraclass correlations (ICCs) to evaluate *agreement* between scores over time by submitting the pre- and post-intervention standardized scores to a two-way mixed effects model for single measurement with absolute agreement. This model factors in both mean-level differences and variance differences between pre- and post-intervention timepoints to determine score reproducibility. ICCs were computed separately for adolescents and parents in each domain.

We used a multipronged approach to interpret agreement. Following Bland and Altman (26), we defined “bias” to reflect a “consistent tendency for one method [rater/time] to exceed the other” (p. 136), which is estimated by the mean difference score. We created Bland Altman plots (25, 26, 34) to visually examine agreement across the full range of observed scores. This involved computing difference scores (T1-T2) and mean scores [(T1+T2)/2] for each participant and plotting the difference scores as a function of the mean scores. We computed the mean difference score, which represents the line of equality (26) - the line on which all points would lie if reports at the two timepoints were the same. We also computed the 95% limits of agreement (mean difference score ± standard deviation*1.96); scores that fall outside of these limits represent potential outliers. These plots visualize whether there is *systematic bias*, meaning that difference scores consistently fall above (i.e., T1 > T2) or below (i.e., T1 < T2) the line of equality. We empirically tested for systematic biases using paired-samples, two-tailed, t-tests comparing mean scores over time within each domain and rater group. When present, the plots also visualize *proportional bias* (i.e., bias that varies with score magnitude (26)). We formally tested for proportional bias by regressing difference scores on the mean scores (26). A significant slope indicated that the magnitude of (dis)agreement between reports over time varied as a function of the score level.

#### 2.4.2 Inter-rater reliability

Inter-rater reliability was evaluated using only pre-intervention data from all dyads in both conditions because it was collected prior to condition assignment. Prior to analyses, we evaluated homogeneity of variance between adolescent and parent scores using Levene’s tests.

To assess linear association, we computed Pearson’s correlations (*r*) between adolescent and parent standardized scores for social skills and problem behaviors. We evaluated agreement between parent and adolescent scores using intraclass correlation; we submitted scores to two-way mixed effects models with absolute agreement. This model treats reporter as a fixed factor with two meaningful levels, not a random sample from a broader population of interchangeable raters. When variance was unmatched, we also conducted an ICC model with consistency, which is less affected by variance mismatches. If the ICC-agreement value was notably lower than the ICC-consistency value, we attributed the differences to variance as opposed to poor inter-rater agreement.

Again, we used Bland Altman plots to visually examine outlier cases, systematic, and *proportional bias*. This involved computing differences scores (parent - adolescent) and mean scores [(parent+adolescent)/2] for each dyad and plotting the difference scores as a function of the mean scores separately for each domain. We also computed the 95% limits of agreement. S*ystematic biases* (e.g., parents reporting fewer social skills than adolescents) were empirically evaluated by comparing group means within each domain (SS, PB) using independent-samples, two-tailed t-tests. Proportional biases were empirically evaluated by regressing difference scores on mean scores; a significant slope indicated that the magnitude of disagreement between parents and adolescents varied systematically with mean score.

## 3 Results

The distributions of standardized scores were all approximately normal per Shapiro-Wilk tests (see **Supplementary Materials**). No scores exceeded acceptable ranges of skew (± 1) or kurtosis (± 1.0).

We characterize the strength of Pearson’s correlations using established benchmarks (35): negligible (0.00 – 0.10), weak (0.11 – 0.39), moderate (0.40 – 0.69), strong (0.70 – 0.89), and very strong (0.90 – 1.00); and ICC strength using benchmarks (36) for poor (< 0.50), moderate (0.50 – 0.75), good (0.75 – 0.90), and excellent (> 0.90) reliability.

### 3.1 Test-retest analyses

#### 3.1.1 Linear association

Adolescents’ self-reported social skills were strongly associated over time, *r*(40) = .730, *p* <.001, *95% CI* [.541,.848], as were their problem behaviors, *r*(40) = .707, *p* <.001, *95% CI* [.507,.835]. Parent scores were moderately associated for both social skills, *r*(40) = .517, *p* <.001, *95% CI* [.245,.714], and problem behaviors, *r*(40) = .579, *p* <.001, *95% CI* [.327,.755].

#### 3.1.2 Agreement

Adolescents exhibited moderate test-retest reliability when reporting social skills, *ICC* = .732, *95% CI* [.546,.849], N = 40, and problem behaviors *ICC* = .699, *95% CI* [.501,.828], N = 40. Parents demonstrated moderate test-retest reliability when reporting social skills, *ICC* = .522, *95% CI* [.252,.716], N = 40, and problem behaviors, *ICC* = .525, *95% CI* [.254,.718], N = 40.

#### 3.1.3 Systematic and proportional bias

There were no systematic biases in adolescents’ reporting of their social skills (T1: *M* = 100.3, *SD* = 16.9; T2: *M* = 100.9, *SD* = 18.4*)*, *t*(39) = -0.30, *p* = .764, or problem behaviors (T1: *M* = 100.3, *SD* = 12.4; T2: *M* = 97.9, *SD* = 12.3), *t*(39) = 0.18, *p* = .860. Similarly, parents showed no systematic biases when reporting social skills (T1: *M* = 81.5, *SD* = 11.1; T2: *M* = 81.2, *SD* = 11.8), *t*(39) = 1.57, *p* = .124. However, parents reported fewer problem behaviors at pre- (T1: *M* = 119.4, *SD* = 10.9) compared to post- (T2: *M* = 124.2, *SD* = 14.5) intervention session, *t*(39) = 2.55, *p* = .015.

Bland Altman plots for control dyads reveal that most difference scores (pre-post) for social skills and problem behavior fell within the 95% limits of agreement (LOA) for adolescents (**Figures 1a, b**) and parents (**Figures 1c, d**), with one adolescent outlier and four parent outliers. Regression analyses revealed no proportional bias in social skills for adolescents, *F*(1, 38) = 0.57, *p* = .453, or parents *F*(1, 38) = 2.22, *p* = .646 (removal of parent outliers did not change this pattern). For problem behavior, there was proportional bias in adolescent scores, *F*(1, 38) = 42.54, *p* <.001, reflecting that those with lower average scores (~80-100) reported an increase over time (i.e., T1 < T2), while those with higher average scores (~110–130) reported a decrease over time (i.e., T1 > T2). There was also proportional bias in parent reports of problem behavior over time, *F*(1, 38) = 4.78, *p* = .035. Parents reporting higher problem behavior on average were more likely to report that these behaviors increased over time (i.e., T1 < T2). However, this bias was reduced when the two outlier scores were removed, *F*(1, 38) = 3.46, *p* = .071.

**Figure 1:**
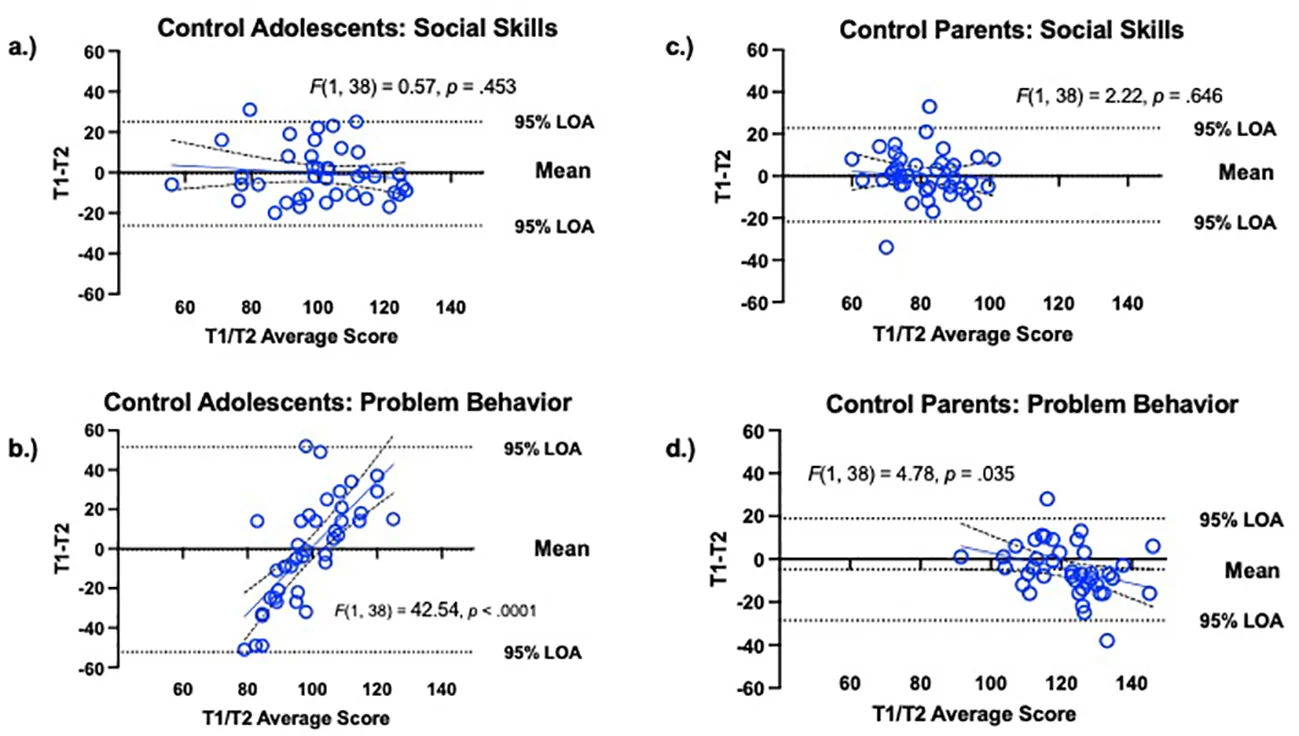
Bland Altman plots comparing adolescent and parent reports of social skills and problem behavior over time. Control adolescents and their parents from both SAGA1 and SAGA2 are combined in this analysis. Control adolescent responses are plotted for social skills **(a)** and problem behavior **(b)**. Control parent responses are plotted for social skills **(c)** and problem behavior **(d)**. Average (over time) and difference scores (T1-T2) were computed separately for each participant in each domain. The Mean line represents the mean difference score and the line of equality. Individual scores above the line represent reveal a bias for scores at T1 to be a higher score than at T2. In contrast, individual scores below the line represent a bias for scores at T1 to be a lower score than at T2. The 95% Lines of Agreement (LOA) represent that confidence intervals around the mean. Scores outside of these lines were identified as outliers and the analyses were conducted both with and without them to evaluate their impact on the results. For each set of data, a linear regression was conducted by regressing difference scores on the mean. A significant effect indicates that the slope is different than zero and implies that there is proportional bias in the difference scores as a function of the mean scores. There was no proportional bias in reports of social skills from either the adolescents or parents. However, there was a proportional bias in adolescents’ problem behavior scores indicating that adolescents with lower mean scores were more likely to report an increase in problem behavior over time (i.e., T1 < T2). Also, adolescents with higher mean scores were more likely to report a decrease in problem behavior over time (i.e., T1 > T2). Parents also exhibited proportional bias in their reports of problem behavior. Parents reporting more problem behavior on average, were more likely to report an increase in these behaviors over time (i.e., T1 < T2).

### 3.2 Inter-rater reliability

#### 3.2.1 Linear Association

Parent and adolescent reports of social skills were significantly, but weakly, associated, *r*(80) = .293, *95% CI* [.078,.482], *p* = .008; however, scores of problem behavior were only marginally associated, *r*(80) = .217, *95% CI* [-.003,.417], *p* = .053.

#### 3.2.2 Agreement

Levene’s tests indicated unequal variance for parent and adolescent reports of social skills, *F*(1, 158) = 12.61, *p* <.001, but not problem behaviors, *F*(1, 158) = .425, *p* = .515. When reporting on social skills, parent-adolescent absolute agreement was significant, but poor, *ICC* = .162, *95% CI* [-.058,.375], *p* = .007. The ICC model with consistency revealed significant and higher agreement, *ICC* = .272, *95% CI* [.057,.463], *p* = .007, which indicates that the low ICC-absolute was likely influenced by variance inhomogeneity. When reporting on problem behavior, parent-adolescent agreement was also significant but poor, *ICC* = .111, *95% CI* [-.066,.299], *p* = .026.

#### 3.2.3 Systematic and proportional bias

There were systematic biases in both domains. Adolescents reported higher social skills (*M* = 96.2, *SD* = 17.3) than did their parents (*M* = 79.4, *SD* = 11.7), *t*(135.3) = 6.87, *p* <.001. Adolescents reported fewer problem behaviors (*M* = 103.2, *SD* = 12.7), than did their parents (*M* = 120.5, *SD* = 12.1), *t*(156) = -8.79, *p* <.001.

The Bland Altman plots (**Figure 2**) illustrate that most of the difference scores (parent – adolescent) fell within the 95% LOA for both social skills and problem behaviors across studies. There was proportional bias in reporting on social skills, *F*(1, 78) = 13.77, *p* = .0004. Among dyads reporting higher average social skills (~90 – 120), adolescents were more likely to report higher scores than parents (parent < adolescent). This pattern held after removing the three outliers. There was no proportional bias in the reports of problem behavior, *F*(1, 78) = 0.21, *p* = .645; removing outliers did not change the pattern of these results.

**Figure 2:**
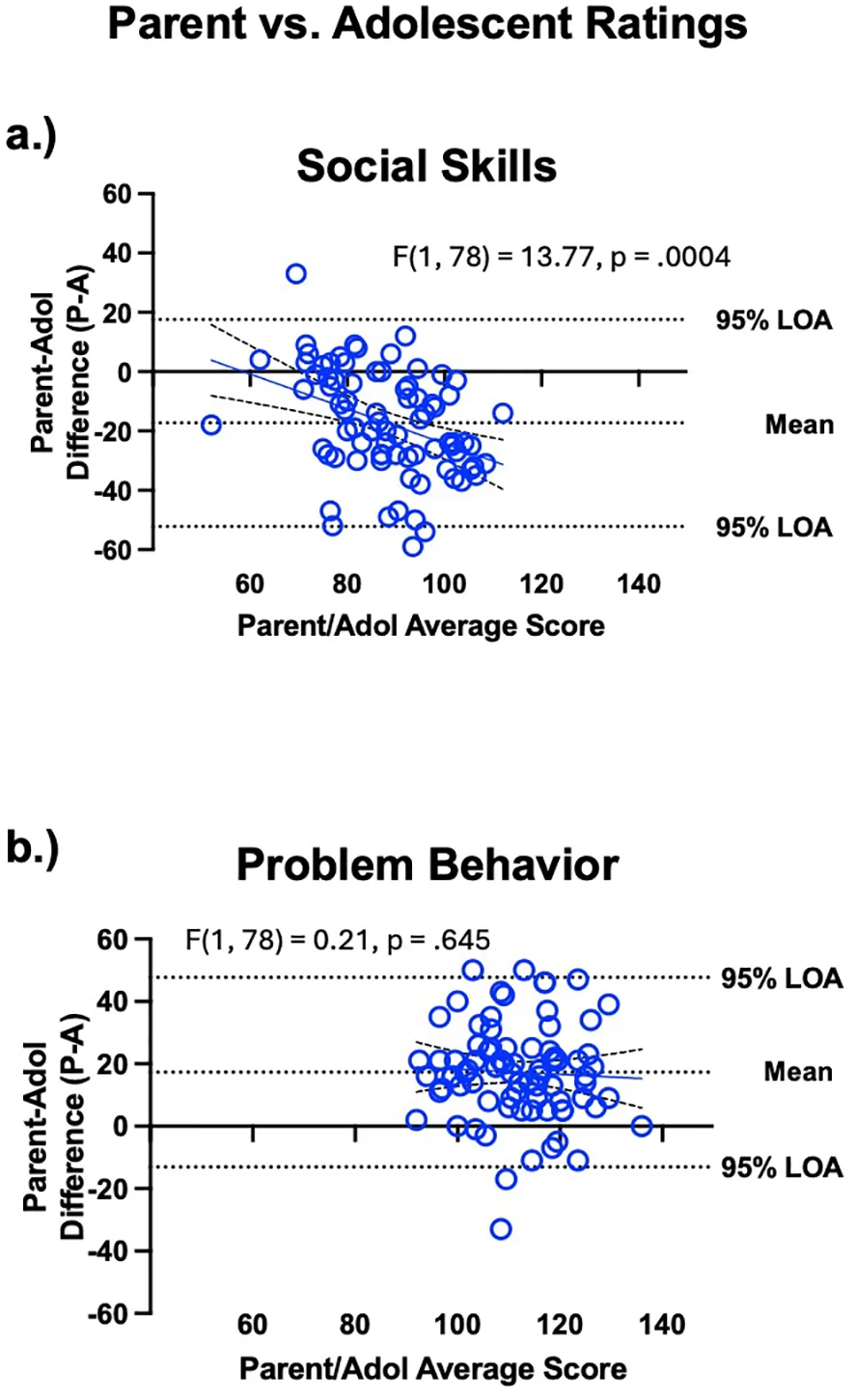
Bland Altman plots of comparing parent and adolescent report of social skills and problem behaviors. All dyads are included from each study. Data were collected at the pre-intervention session prior to condition assignment, so control and intervention data are combined. Parent-adolescent average scores and difference scores were computed separately for each dyad in each domain. Responses for social skills **(a)** and problem behavior **(b)** are plotted and analyzed separately. The Mean represents the mean difference score and the line of equality. Individual scores above the line represent a bias for parent scores to be a higher than adolescent scores. In contrast, scores below the line represent a bias for parent scores to be a lower score than adolescent scores. The 95% Lines of Agreement (LOA) represent that confidence intervals around the mean. Scores outside of these lines were identified as outliers and the analyses were conducted both with and without these scores to evaluate the impact of the outliers on the results. For each set of data, a linear regression was conducted by regressing difference scores on the mean. A significant effect indicates that the slope is different than zero and implies that there is proportional bias in the difference scores as a function of the mean scores. There was significant proportional bias in the reports of social skills, which was unchanged when the outliers were removed; it reflects that in dyads reporting higher average social skills (~90-120), adolescents were more likely to report higher scores than their parents. In contrast, there was no proportional bias in reports of problem behavior, even when the outlier scores were removed.

## 4 Discussion

The current study examined autistic adolescent-parent informant discrepancies on reports of adolescents’ social skills and problem behaviors. We examined whether low test-retest reliability in either adolescent or parent reports might contribute to high informant discrepancies between autistic adolescents and their parents. Across two RCTs, autistic adolescents and parents reported on adolescents’ social skills and problem behaviors using the SSIS approximately 2–3 months apart. Test-retest reliability estimates were focused on the dyads assigned to control conditions in which social skills and problem behaviors were expected to remain stable over time. Finally, we used baseline data to evaluate informant discrepancies, combining adolescent and parent reports across conditions and studies given that these data were collected prior to randomization. In all analyses, reliability and agreement were examined separately for social skills and problem behaviors.

### 4.1 Reliability of responses over time

There is very little data evaluating the stability of autistic adolescents’ reports of their own social behavior. Evaluating stability of responses helps reduce concerns about random, unorganized, inconsistent reporting from autistic adolescents. In fact, we observed moderate reliability in adolescent self-reported social skills and moderate reliability in their reporting of problem behavior. The stability we observed in autistic adolescent reports of their own behavior is comparable to that reported in a meta-analysis of non-autistic adolescents (2).

There was no systematic or proportional bias in autistic adolescents’ reporting of their social skills. This was evident from the direct comparison of pre- and post- scores and in the Bland Altman plots illustrating the full range of T1-T2 difference scores. In contrast, we observed bias in adolescent’s reporting of problem behaviors. There was no systematic bias (i.e., T1=T2), but the reporting did exhibit a proportional bias. Specifically, autistic adolescents reporting a lower average score for problem behaviors (~80-100) were more likely to report an *increase* in these behaviors over time (i.e., T1 < T2). In contrast, autistic adolescents reporting a higher average problem behavior score (~110-130) were more likely to report a *decrease* in these behaviors over time (i.e., T1 > T2). Although this proportional bias could reflect regression to the mean over time, a similar regression to the mean was not observed in adolescent reports of their social skills. Also, the proportional bias cannot be explained by factors like fatigue or practice effects that would have been detected in the assessment of systematic bias. Finally, this proportional bias was present in each of the study specific analyses as well (see **Supplementary Materials**), indicating that it was replicated and unaffected by the differences between studies. Together, these results challenge the notion that autistic adolescents are incapable of providing reliable, meaningful reports about their own behavior. Instead, they suggest that autistic adolescents who are verbal, have a minimal reading level, and an average full-scale IQ provide good stability in their reports of their own social skills over time. They also provide evidence of individual differences in this stability, particularly in the way autistic adolescents report on their problematic behavior, which lead to novel questions about how autistic adolescents perceive, experience, and report on their own challenging behavior.

In terms of the stability of parent reports of autistic adolescent behavior, as expected, we observed moderate test-retest reliability for parent reports of adolescents’ social behavior and problem behaviors. There were no systematic or proportional biases in parents’ reports of social skills, but there was a different pattern in their reports of problem behavior. Parents systematically reported fewer problem behaviors over time (i.e., T1 > T2). Also, there was a proportional bias in parent responses - parents who reported higher average problem behaviors (~120-140) were most likely to report an increase in these behaviors over two time points (i.e., T1 < T2). However, conclusions about this proportional bias must be tempered by the finding that removing the outlier scores, minimized the bias to be non-significant. Also, the effect was only present in the SAGA2 study specific analyses. Therefore, it may reflect a more specific cohort effect that is less generalizable.

It is also important to acknowledge that the control participants in SAGA1 and SAGA2 had different experiences, which may have influenced expectations about behavior change over time, particularly from parents. SAGA1 control dyads were randomized to treatment-as-usual and unblinded to not receiving the psychosocial intervention, which may have limited their expectations of change. SAGA2 control dyads were randomized to an active control game but blinded to this assignment, potentially producing stronger expectations of change in adolescent behavior. Study-specific analyses (see **Supplementary Materials**) partially support this: parent reliability was weaker in SAGA2 than in SAGA1 for both social skills and problem behavior, consistent with stronger expectancy bias among SAGA2 parents, while adolescent reliability was moderate across both studies. However, the proportional bias in reporting on problem behavior is inconsistent with this interpretation; it suggests parents are reporting more (not less) problem behavior over time, especially for those adolescents with higher average scores.

Together, these findings suggest that autistic adolescent and parent reporting of social skills is relatively stable over time (across 2-to-3-month intervals); however, both adolescents and parents perceive changes in problem behaviors over time. Adolescents perceive these behaviors to be decreasing over time, especially if they report higher levels of problem behavior; whereas parents perceive problem behaviors to be increasing over time, especially if they report high numbers of these behaviors in their adolescents. These findings suggest that adolescents and parents perceive quite different patterns of change in problem behavior during adolescence. Future longitudinal work will need to replicate and interpret these findings more directly, perhaps using multiple measures assessing problem behaviors in adolescents.

### 4.2 Autistic adolescent-parent informant discrepancies

Despite finding moderate stability in autistic adolescents’ behavioral reports over time, at baseline, adolescent and parent reports of social skills and problem behaviors were positively, but weakly, associated with poor agreement across domains. These findings are consistent with prior work reporting high autistic adolescent-parent informant discrepancies on other measures of social skills and autistic traits (7, 37).

The Bland Altman plots suggest systematic biases contributed to these poor levels of association and agreement. As predicted and consistent with prior work (14, 15, 17, 18), autistic adolescents rated their social skills more favorably than did their parents. Notably, autistic adolescents’ average scores for social skills fell within the average range for non-autistic adolescents across both studies (22). Adolescents also reported systematically fewer problem behaviors than did their parents.

There were proportional biases in the reporting of social skills that are reflected on the Bland Altman plots: adolescent-parent discrepancies increased as social skills increased, with adolescents reporting higher scores than parents. To our knowledge, this pattern has not been previously reported and is consistent with the notion that autistic adolescents, but perhaps not their parents, are able to consider increasing situational specificity of their social skills across multiple contexts. Future work will need to evaluate and confirm this possible explanation for this finding.

### 4.3 Limitations and future directions

The samples from these RCTs were relatively small and demographically homogenous: all participants were verbal with average full-scale IQs and were able to complete the study protocol. Most participants identified as white males and parent reports were primarily provided by mothers. Therefore, we could not investigate whether participant or parent gender/sex at birth influenced the direction or magnitude of informant discrepancies. Given emerging evidence that such discrepancies may vary by gender/sex at birth of both the adolescent and informant (37–39), future studies should recruit more diverse samples to directly examine these effects. It is also important to adapt methods to be inclusive for use with minimally speaking or intellectually disabled adolescents, whose reports may reflect different reliability or informant discrepancies.

Second, the study did not include a non-autistic comparison group, therefore, we cannot determine whether and to what extent the observed findings reflect autism-specific adolescent developmental factors or patterns related to the development of adolescence more generally. Future work should include autistic and non-autistic adolescents within the same design to directly compare the magnitude, direction, and contributing mechanisms of informant discrepancies.

Finally, a core assumption of the intraclass correlation approach is that different raters make reports about identical observations; however, our findings challenge this assumption about the SSIS. Adolescents and parents receive different instructions that might magnify the situational specificity of adolescent behavior, encouraging adolescents to draw from a wider range of contexts to rate their behavior in a trait-like way, while parents must rate observable behavior in perhaps more limited contexts. A critical next step is to use emerging methods to evaluate these informant discrepancies and the underlying rater differences. For example, the CONTEXT model (6) could be used to evaluate how the unique variance in adolescent and parent reports differentially predict a third variable (e.g., adaptive functioning, peer acceptance, friendship qualities, well-being) to distinguish sources of common/unique variance, rater biases, and measurement error. Finally, including peer raters and independent behavioral ratings of peer interactions (40) could inform understanding about the informant discrepancies and situational specificity of autistic adolescents’ behavior.

### 4.4 Implications and conclusion

Our findings suggest that autistic adolescents provide reliable self-reports of their social skills and problem behavior. Therefore, including autistic adolescents’ self-reports alongside parent or peer reports may provide unique and critical information about social functioning, and problem behavior in particular, during adolescence in autism. This has clinical – not just methodological stakes – if adolescents can access aspects of their social experience (i.e., friendships and interactions occurring outside of the home) that parents and other observers cannot directly witness, then excluding their perspective means researchers and clinicians are missing important information relevant to understanding social behavior in autism, not simply losing precision in discrepancy measurements. Ultimately, including autistic adolescent reports will critically inform models of social transition and adolescent development in autism.

## Data Availability

The datasets presented in this study can be found in online repositories. The names of the repository/repositories and accession number(s) can be found below: National Institutes of Health Data Archive (NDA) - https://nda.nih.gov/edit_collection.html?id=2423.
