# Supplementary material for: Ask autistic adolescents too: informant discrepancies and test-retest reliability in social behavior
Source: Front Psychiatry. 2026 Sep 21;17:1819507. doi: 10.3389/fpsyt.2026.1819507 (PMC13635734; doi:10.3389/fpsyt.2026.1819507)
Supplement: Supplementary file 1 [file SupplementaryFile1.docx]

**Supplementary Materials**

|  | **SAGA1** | | **SAGA2** | |  |
| --- | --- | --- | --- | --- | --- |
|  | **Control** | **Intervention** | **Control** | **Intervention** | **p** |
| ***N*** | 20 | 19 | 20 | 21 |  |
| **Age in Months** | 163.80 (31.88) | 167.68 (33.51) | 164.05 (24.84) | 172.05 (29.75) | *.797* |
| **Sex -** N (% ) male | 17 (85%) | 15 (78.9%) | 19 (95%) | 16 (76%) | .*320* |
| **IQ** |  |  |  |  |  |
| Full Scale IQ | 99.65 (13.42) | 99.63 (16.67) | 99.65 (16.34) | 104.10 (16.43) | .745 |
| Verbal IQ | 97.95 (18.52) | 97.11 (15.78) | 97.20 (19.20) | 99.33 (20.11) | .979 |
| Non-Verbal | 100.60 (10.82) | 101.95 (14.66) | 101.80 (16.36) | 107.24 (15.17) | .460 |
| **Clinical Scores** |  |  |  |  |  |
| ADOS | 13.80 (4.24) | 13.16 (5.05) | --- | --- | .669 |
| SCQ | --- | --- | 20.55 (7.76) | 20.14 (4.71) | .839 |

**Table S1: Demographic and Clinical Characteristics of Participants as a Function of Study**

Note: Age, IQ, and clinical scores are presented as mean (standard deviation; SD). Sex is presented as the total number (percentage) of male participants; remaining participants identified as female except for one SAGA2 intervention participant who identified as non-binary. Clinical diagnostic characteristics differed across studies due to the COVID-19 pandemic; SAGA1 utilized in-person eligibility assessments with the ADOS-2, while SAGA2 eligibility assessments were conducted remotely using the Social Communication Questionnaire (SCQ) and clinician interview. ANOVAs confirmed no significant demographic or clinical differences between groups; p-values of these group comparisons within each study are reported in the final column. SAGA1 and SAGA2 participants were independent samples with no overlap across studies. SAGA1-NCT02968225, SAGA2-NCT03690661.

**Results**

*Testing normality of data contributing to the test-retest analyses in the full manuscript*

Table S2 shows that all the data contributing to the test-retest analyses was approximately normally distributed, as indicated by the acceptable ranges of skew and kurtosis (± 1) and the non-significant Shapiro Wilk tests.

**Table S2. Normality tests on data contributing to the test-retest analyses**

| **Domain** | **Time-Rater** | **Mean [95% CI]** | **Skew** | **Kurtosis** | **Shapiro Wilk** |
| --- | --- | --- | --- | --- | --- |
| Social Skills | T1 Participant | 100.3 [94.9, 105.7] | -.722 | -.127 | *W*(40) = .949, *p* = .068 |
|  | T2 Participant | 100.9 [95.0, 106.8] | -.289 | -.133 | *W*(40) = .965, *p* = .256 |
|  | T1 Parent | 81.5 [78.0, 85.1] | -.132 | .060 | *W*(40) = .984, *p* = .840 |
|  | T2 Parent | 81.2 [77.4, 85.9] | -.134 | -.833 | *W*(40) = .975, *p* = .519 |
| Problem Behavior | T1 Participant | 100.3 [96.3, 104.2] | .063 | -.865 | *W*(40) = .971, *p* = .389 |
|  | T2 Participant | 97.9 [94.0, 101.9] | .664 | .577 | *W*(40) = .965, *p* = .257 |
|  | T1 Parent | 119.4 [115.9, 122.8] | .021 | .851 | *W*(40) = .983, *p* = .809 |
|  | T2 Parent | 124.2 [119.6, 128.9] | -.098 | -.569 | *W*(40) = .977, *p* = .568 |

*Test-Retest Analyses for each study (SAGA1, SAGA2) separately*

The control groups in the two studies had slightly difference experiences; in SAGA1 the control group was a treatment as usual group, however, in SAGA 2 the control group played an active control game for three months. Therefore, we computed the test-retest analyses on each study group separately to provide additional information about the patterns of findings.

*Linear Association*. In SAGA1, adolescent self-reported skills were moderately associated over time, *r*(18) = .594, *p* = .006, *95% CI* [.206, .812]; however, their self-reported problem behaviors were strongly associated over time, *r*(18) = .768, *p* <.001, *95% CI* [.493, .903]. Parent scores exhibited a different pattern; they were moderately associated for both social skills, *r*(18) = .635, *p* = .003, *95% CI* [.268, .841], and problem behaviors, *r*(18) = .591, *p* = .006, *95% CI* [.202, .819].

In SAGA2, adolescents self-reported social skills were strongly associated over time, *r*(18) = .725, *p* <.001, *95% CI* [.415, .884], and problem behaviors were moderately associated, *r*(18) = .674, *p* =.001, *95% CI* [.329, .860]. Parent scores were weakly associated for social skills, *r*(18) = .290, *p* = .215, *95% CI* [-.175, .649], and moderately associated for problem behaviors, *r*(18) = .636, *p* = .003, *95% CI* [.269, .841].

*Agreement.* In terms of the agreement of reporting social skills*,* adolescents in SAGA1 (n = 20), demonstrated moderate test-retest reliability, *ICC* = .589, *95% CI* (.221, .812), as did their parents, *ICC* = .635, *95% CI* (.276, .838). In SAGA2 (n = 20), adolescents demonstrated moderate reliability *ICC* = .729, *95% CI* (.434, .883), and parents demonstrated poor reliability, ICC = .282, *95% CI* (-.179, .639), when reporting on adolescent social skills over time.

In terms of the stability of reporting problem behavior*,* adolescents in SAGA1 demonstrated good reliability, *ICC* = .759, *95% CI* (.481, .897), as did their parents, *ICC* = .589, *95% CI* (.199, .816). In SAGA2, adolescents demonstrated moderate reliability, *ICC* = .636, *95% CI* (.283, .838), but parents demonstrated poor reliability, *ICC* = .457, *95% CI* (-.018, .756), when reporting on adolescent problem behavior over time.

*Systematic and Proportional Bias*. For SAGA1 (n = 20) control dyads, there were no systematic biases in adolescents’ reporting of their social skills at the pre- (*M* = 106.5, *SD* = 13.9) versus post- (*M* = 109.7, *SD* = 13.3*)*, intervention sessions, *t*(19) = -1.19, *p* = .250; or in their reports of problem behaviors at the pre- (*M* = 96.4, *SD* = 10.4) versus post- (*M* = 96.3, *SD* = 13.0) intervention sessions, *t*(19) = 0.54, *p* = .958. Similarly, there were no systematic biases in parents’ reporting of their adolescents’ social skills at the pre- (*M* = 84.0, *SD* = 12.9) versus post- (*M* = 85.3, *SD* = 10.9) intervention sessions, *t*(19) = -0.55, *p* = .589, or in the problem behaviors at the pre- (*M* = 116.6, *SD* = 13.7) compared to post- (*M* = 118.1, *SD* = 11.5) intervention session, *t*(19) = -.600, *p* = .556.

Among SAGA2 control dyads, there was no systematic bias in adolescents’ reporting of their social skills across the pre- (*M* = 94.1, *SD* = 17.7) and post- (*M* = 92.1, *SD* = 18.9) intervention sessions, *t*(19) = 0.66, *p* =.520; however, they tended to be biased in reporting more problem behaviors in the pre- (*M* = 104.2, *SD* = 13.2) compared to post- (*M* = 99.6, *SD* = 11.7) intervention sessions, *t*(19) = 2.02, *p* = .057. Similarly, there was no systematic bias in parents reporting of adolescent social skills across the pre- (*M* = 79.1, *SD* = 8.5) and post- (*M* = 77.2, SD = 11.5) intervention sessions, *t*(19) = 0.70, *p* = .494. However, parents were systematically biased in reported more problem behaviors in the pre- (*M* = 120.6, *SD* = 9.9) compared to post- (*M* = 130.4, *SD* = 14.8), *t*(19) = -3.82, *p* =.001, intervention sessions.

Finally, the Bland Altman plots for SAGA1 (Figure S1) control dyads reveal that most difference scores (pre-post) for social skills and problem behavior fall within the 95% lines of agreement (LOA) for adolescents (Figs S1a, b) and parents (Figs S1c, d). There were two outlier scores (i.e., outside the 95% LOA) among the adolescents and two among the parents. Removal of these outliers did not impact the pattern of results. The regression analyses revealed no proportional bias for either adolescents, *F*(1, 18) = 0.06, *p* = .806, or parents *F*(1, 18) = 0.84, *p* = .372, in their reporting of social skills. In contrast, for problem behavior adolescents (Fig 1b) exhibit significant proportional bias, *F*(1, 18) = 35.67, *p* < .001, indicating that participants with lower average scores reported an increase in problem behaviors over time (i.e., negative pre-post difference scores). Parents did not exhibit proportional bias in their scores of problem behavior over time, *F*(1, 18) = 0.04, *p* = .836.

**Figure S1. Bland Altman Plots Comparing Control Adolescent and Control Parent Reports of Social Skills and Problem Behavior Over Time in SAGA1**

Note: Control adolescent responses are plotted for social skills (a) and problem behavior (b). Control parent responses are plotted for social skills (c) and problem behavior (d). Average (over time) and difference scores (T1-T2) were computed separately for each participant in each domain. The Bias represents the mean difference scores. Individual scores above the Bias represent bias for T1 to be a higher score than T2. In contrast, individual scores below the Bias represent bias for T1 to be a lower score than T2. The 95% Lines of Agreement (LOA) represent that confidence intervals around the Bias. Scores outside of these lines were identified as outliers and the analyses were conducted both with and without these scores to evaluate the impact of the outliers on the results. For each set of data, a linear regression was conducted by regressing difference scores on the mean scores. A significant effect indicates that the slope is different than zero and implies that there is proportional bias in the difference scores as a function of the mean scores. There was no proportional bias for the control parent scores or adolescent social skills over time. However, there was a proportional bias in adolescents’ problem behavior scores.

The Bland Altman plots for SAGA2 (Figure S2) control dyads reveal that most difference scores (pre-post) for social skills and problem behavior fall within the 95% LOA for adolescents (Figs S2a, b) and parents (Figs S2c, d). Among the SAGA2 control dyads, there were two adolescent and two parent outlier scores. The regression analyses revealed no significant proportional bias for either adolescents, *F*(1, 18) = 0.15, *p* = .703, or parents, *F*(1, 18) = 1.18, *p* = .195, in their reporting of social skills. However, removal of the adult outlier led to a significant proportional bias, *F*(1, 17) = 5.03, *p* = .040, indicating that parents reporting higher social skills on average in their adolescent also reported a reduction in social skills over time (i.e., negative pre-post difference score). For problem behaviors, there were proportional biases in both the adolescent, *F*(1, 18) = 13.10, *p* = .002, and parent, *F*(1, 18) = 5.25, *p* = .034, reports. Removal of the adolescent (N = 1) and parent (N = 1) outliers did not change the pattern of findings. Among adolescents, those who reported higher problem behavior scores on average, reported a decrease in the behaviors over time (i.e., positive pre-post difference score). In contrast, parents reporting higher problem behaviors on average, reported an increase in the behaviors over time (i.e., negative pre-post difference score).

**Figure S2. Bland Altman Plots Comparing Control Adolescent and Control Parent Reports of Social Skills and Problem Behavior Over Time in SAGA2**

Note: Control adolescent responses are plotted for social skills (a) and problem behavior (b). Control parent responses are plotted for social skills (c) and problem behavior (d). Average scores (over time) and difference scores (T1-T2) were computed separately for each participant in each domain. The Bias represents the mean difference scores. Individual scores above the Bias represent bias for T1 to be a higher score than T2. In contrast, individual scores below the Bias represent bias for T1 to be a lower score than T2. The 95% Lines of Agreement (LOA) represent that confidence intervals around the Bias. Scores outside of these lines were identified as outliers and the analyses were conducted both with and without these scores to evaluate the impact of the outliers on the results. For each set of data, a linear regression was conducted by regressing the difference scores on the mean scores. A significant effect indicates that the slope is different than zero and implies that there is proportional bias in the difference scores as a function of the mean scores. There was no proportional bias for adolescent or parent social skills over time. However, there were proportional biases in both problem behavior scores for adolescents and parents. The proportional bias for parent problem behavior was influenced by the outlier data point; eliminating it from the analysis led to an insignificant result.

*2.3* *Testing normality of data contributing to the inter-rater analyses in the full manuscript*

Table S3 shows that all the data contributing to the inter-rater analyses was approximately normally distributed, as indicated by the acceptable ranges of skew and kurtosis (± 1) and the non-significant Shapiro Wilk tests.

**Table S3. Normality tests on data contributing to the inter-rater analyses**

| **Domain** | **Time-Rater** | **Mean [95% CI]** | **Skew** | **Kurtosis** | **Shapiro Wilk** |
| --- | --- | --- | --- | --- | --- |
| Social skills | T1 Participant | 98.8 [93.0, 101.7] | -.321 | -.585 | *W*(80) = .971, *p* = .062 |
|  | T1 Parent | 79.5 [76.9, 82.1] | -.443 | .438 | *W*(80) = .977, *p* = .157 |
| Problem Behavior | T1 Participant | 103.2 [100.4, 106.0] | .183 | -.553 | *W*(80) = .984, *p* = .434 |
|  | T1 Parent | 120.5 [117.8, 123.2] | -.023 | -.207 | *W*(80) = .990, *p* = .822 |

*2.4 Inter-rater Analyses for each study (SAGA1, SAGA2) separately*

The two studies were conducted in different temporal contexts; SAGA1 was conducted prior to the COVID-19 global pandemic, whereas SAGA 2 was conducted during the pandemic. Therefore, we computed the inter-rater analyses on each study group separately to provide additional information about the patterns of findings.

*2.4.1. Association.* In SAGA1, adolescent and parent reports were weakly associated at the pre-intervention session for social skills, *r*(37) = .353, *p* =.028, *95% CI* [.042, .601], and moderately associated for problem behaviors, *r*(37) = .479, *p* = .002, *95% CI* [.193, .690]. Similarly, in SAGA2, adolescent and parent report was weakly associated at baseline for social skills *r*(39) = .302, *p* = .055, *95% CI* [-.006, .558], but not associated for problem behaviors *r*(39) = -.112, *p* = .485, *95% CI* [-.406, .202].

*2.4.2. Agreement.* Levene’s tests of homogeneity indicated that parent and adolescent reports of both social skills, *F*(1, 76) = 0.67, *p* = .42, and problem behaviors, *F*(1, 76) = 0.62, *p* = .43, in SAGA1 exhibited comparable variance. Also, in SAGA 2 variance was comparable for problem behavior, *F*(1, 80) = 1.99, *p* = .16, however, it was unmatched for social skills, *F*(1, 80) = 22.40, *p* < .001. As a result, we also ran the ICC model with consistency to evaluate to contributions of the unmatched variance.

In SAGA1 (n = 40), inter-rater reliability for reporting on adolescent social skills, *ICC* = .158, *95% CI* [-.089, .431], and problem behavior, *ICC* = .209, *95% CI* [-.096, .521], was poor. In SAGA2 (n = 41), inter-rater reliability was also poor for adolescent social skills, *ICC* = .192, 95% *CI* [-.067, .444], and problem behaviors, *ICC* = -.063, 95% *CI* [-.229, .152]. Given the lack of variance match for social skills reporting in SAGA2, the consistency model was run; it indicated poor (but higher) agreement, *ICC* = .253, 95% *CI* [-.055, .518]. These findings suggest that the poor reliability in reporting of adolescent social skills was likely influenced by the variance inhomogeneity between parent and adolescent scores.

*2.4.3. Systematic and Proportional Bais.* There were systematic biases in both the social skills and problem behaviors. Across both studies adolescents as a group reported higher social skills and fewer problem behaviors than did their parents. In SAGA1 (n = 39) adolescents’ self-reported social skills (*M* = 103.5, *SD* = 15.9) were higher than parent reports (*M* = 79.3, *SD* = 14.2), *t*(1, 76) = 7.09, *p* <.001. Adolescents’ self-reported problem behaviors (*M* = 99.4, *SD* = 12.0) were lower than parent reports (*M* = 118.8, *SD* = 14.5), *t*(1, 76) = -6.45, *p*<.001. In SAGA2 (n = 41), adolescent self-reported social skills (*M* = 90.5, *SD* = 16.2) were higher than parent-report (*M*= 79.7, *SD* = 8.8), *t*(1, 80) = 3.73, *p* <.001. Adolescent self-reported problem behaviors (*M* = 106.9, *SD* = 12.5) were lower than parents (*M* = 121.4, *SD* = 10.6), *t*(1, 80) = -5.67, *p* <.001.

The Bland Altman plots for SAGA1 and SAGA2 are represented in Figure S3. Most of the difference scores (parent – adolescent) fell in the 95 LOA for both social skills and problem behaviors across studies. In SAGA1, there was no proportional bias for social skills, *F*(1, 37) = 0.54, *p* = .464, or problem behaviors, *F*(1, 37) = 0.75, *p* = .393. However, in SAGA2 there was a proportional bias in social skills, *F*(1, 38) = 21.54, *p* < .001, indicating that in dyads who reported higher average social skill scores, adolescents reported the higher score (i.e., negative parent-adolescent difference score). Removal of the two outlier dyads did not change the pattern of results. There was no proportional bias in the reporting of problem behavior in SAGA2, *F*(1, 38) = 1.28, *p* = .275. Removal of the outlier dyad did not change the pattern of results.

**Figure S3. Bland Altman Plots of Comparing Parent and Adolescent Report of Social Skills and Problem Behaviors in SAGA1 and SAGA2**

Note: All dyads are included from each study. Data were collected at the pre-intervention session, so control and intervention data are combined. SAGA1 responses for social skills (a) and problem behavior (b); SAGA2 responses for social skills (c) and problem behavior (d). Parent-adolescent average scores and difference scores were computed separately for each dyad in each domain. The Bias represents the mean difference scores. Individual scores above the Bias represent bias for parent scores to be a higher than adolescent scores. In contrast, individual scores below the Bias represent bias for parent scores to be a lower score than adolescent scores. The 95% Lines of Agreement (LOA) represent that confidence intervals around the Bias. Scores outside of these lines were identified as outliers and the analyses were conducted both with and without these scores to evaluate the impact of the outliers on the results. For each set of data, a linear regression was conducted by regressing the difference scores on the mean score. A significant effect indicates that the slope is different than zero and implies that there is proportional bias in the difference scores as a function of the mean scores. There was no proportional bias for social skills or problem behavior in SAGA1. In contrast, there was a proportional bias in social skills (c), but not problem behavior (d), scores in SAGA2. The two outlier scores in SAGA2 social skills did not impact the pattern of results when excluded from the analyses.
